# Case Report: Successful multimodal management of a lethal cyanide poisoning – insights into the role of early blood purification and potential bedside monitoring indicators

**DOI:** 10.3389/fphar.2026.1837748

**Published:** 2026-06-15

**Authors:** Hang Xie, Yue Zhang, Xin Luo, Jiaqi Xu, Chao Chen, Yujia Qu, Lijun Wang

**Affiliations:** Department of Emergency Medicine, Tianjin Medical University General Hospital, Tianjin, China

**Keywords:** blood purification, central venous carbon dioxide partial pressure, central venous oxygen partial pressure, cyanide poisoning, lactate, potassium ferrocyanide

## Abstract

This article reports a rare and lethal case of cyanide poisoning, aiming to explore its early diagnosis and successful treatment strategies. Upon admission, the patient presented with cardiac and respiratory arrest, deep coma, and severe metabolic acidosis. The core resuscitation measures included early mechanical ventilation, vasoactive drug support, bicarbonate therapy, and the early initiation of continuous blood purification as part of a multimodal strategy. We observed that a combination of elevated central venous partial pressure of carbon dioxide (PvCO_2_) and elevated lactate provided potential bedside clues for early suspicion of cyanide poisoning. During treatment, a rise in PvCO_2_ accompanied by a decrease in lactate was noted, which may serve as a hypothesis-generating observation for treatment response, acknowledging the influence of multiple concurrent interventions. The patient recovered substantially after comprehensive treatment. This case provides valuable experience for the treatment of cyanide poisoning.

## 1 Introduction

Cyanide poisoning has been involved in various historical events, including gas chambers, warfare, suicide, or homicide (Hendry-Hofer et al., 2019). Due to its lethality and the non-specificity of early symptoms, rapid diagnosis and timely intervention are crucial for saving lives. Here we report a case of a 46-year-old male who presented with cardiac arrest, deep coma, and severe metabolic acidosis after suspected cyanide ingestion. Laboratory tests revealed a blood pH of 6.57, lactate >20 mmol/L, and markedly elevated central venous oxygen partial pressure (PvO_2_). The patient was successfully resuscitated with early continuous blood purification. This report focuses on the early diagnostic clues and treatment monitoring indicators derived from this case.

## 2 Case description

At 10:00 a.m. on 17 January 2025, a 46-year-old male patient was sent to a local hospital after being found unconscious by a passerby. During evaluation and treatment, the patient suddenly developed convulsions, bradycardia, and bradypnea. After cardiopulmonary resuscitation, bag-valve-mask ventilation, and administration of dopamine and epinephrine, his condition temporarily stabilized partially. Family members found a purchase record for “Potassium Ferrocyanide” on his phone, leading to a high suspicion of poisoning. He was urgently transferred to our hospital’s emergency department at 11:45 a.m. the same day. His blood pressure was 60/40 mmHg, heart rate 60 beats/min, oxygen saturation 70%. He was in a coma, exhibiting agonal respiration, with bilaterally dilated and fixed pupils unresponsive to light, and cold skin. Immediate endotracheal intubation with mechanical ventilator support, gastric lavage via nasogastric tube, and aggressive circulatory support were initiated.

Laboratory tests revealed: White blood cell count 22.46*10^9^/L, Red blood cell count 4.50*10^12^/L, Hemoglobin 139 g/L, Platelet count 304*10^9^/L, Neutrophil percentage 44.7%, Creatinine 193 μmol/L, Uric acid 718 μmol/L, Amylase 774 U/L, Blood ammonia 478.0 μmol/L, Plasma D-dimer quantitative 4,616 ng/mL. Blood gas analysis showed pH 6.57, PaCO_2_ 63.5 mmHg, PaO_2_ 117 mmHg(FiO_2_ 100%), Lactate >20 mmol/L. The elevated serum creatinine (193 μmol/L) and uric acid (718 μmol/L) on admission reflected acute kidney injury, likely multifactorial due to cardiogenic shock, tissue hypoxia, and possible direct tubular toxicity of cyanide. The marked hyperammonemia (478 μmol/L) has been described in cyanide poisoning as a result of impaired hepatic mitochondrial function and urea cycle disruption. Elevated amylase (774 U/L) suggested mild pancreatic injury, possibly from hypoperfusion or direct toxin effect. The high D-dimer (4,616 ng/mL) indicated activation of coagulation and fibrinolysis, consistent with systemic inflammatory response and tissue necrosis following cardiac arrest. These laboratory derangements resolved completely by discharge, supporting their acute, reversible nature in the setting of severe cyanide toxicity.

**FIGURE 1:**
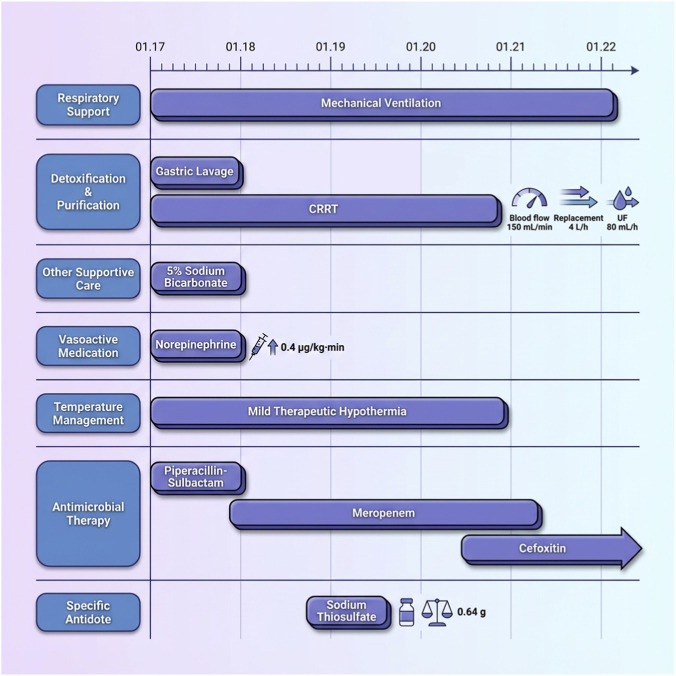
Treatment Timeline of the Cyanide Poisoning Patient. The flowchart illustrates the sequence of therapeutic interventions from hospital admission (Day 1) to discharge (Day 7). Key interventions include mechanical ventilation, continuous renal replacement therapy (CRRT) initiated within 1 h of ICU admission with parameters: blood flow rate 150 mL/min, replacement fluid rate 4 L/h, and ultrafiltration 80 mL/h; vasoactive support with norepinephrine (0.4 μg/kg/min); administration of sodium thiosulfate (0.64 g); and antimicrobial therapy adjustments.

After being urgently admitted to the Emergency Intensive Care Unit, the patient’s blood pressure further dropped to 50/30 mmHg. Upon admission to the Emergency Intensive Care Unit and after initial resuscitation including fluid and vasoactive support, repeat arterial blood gas (FiO_2_ 100%) showed: pH 6.68, PaCO_2_ 45.4 mmHg, PaO_2_ 279.4 mmHg, lactate still >20 mmol/L. Central venous blood gas obtained simultaneously at the latter time point showed: PvO_2_ 116.3 mmHg, PvCO_2_ 44.5 mmHg. While administering 5% sodium bicarbonate to correct acidosis, bedside continuous venovenous hemofiltration was rapidly initiated (blood flow rate: 150 mL/min, replacement fluid rate: 4 L/h, ultrafiltration: 80 mL/h). Following approximately 3 h of active resuscitation, the patient’s vital signs stabilized. Subsequently, cyanide ions were detected in the blood and gastric fluid at concentrations of 0.3 μg/mL and 3.6 μg/mL, respectively, using gas chromatography-mass spectrometry, confirming the diagnosis of acute cyanide poisoning. Sodium thiosulfate (0.64 g) was administered intravenously as an antidote. The dose of sodium thiosulfate (0.64 g) was approximately 5% of the standard recommended dose (typically 12.5 g for adults). The reasons for this reduced dose were multifactorial. First, hydroxocobalamin—the preferred first-line antidote for suspected cyanide poisoning—was not available in our hospital. Second, the standard 12.5 g dose of sodium thiosulfate was also out of stock at the time, and a supply had to be transferred from another hospital. During the waiting period for the antidote to arrive, the patient’s hemodynamic status and metabolic acidosis gradually improved with ongoing continuous renal replacement therapy and supportive care. By the time the antidote became available, the patient’s vital signs had largely stabilized. Therefore, only a low dose (0.64 g) of sodium thiosulfate was administered as a cautious adjunct, rather than as a definitive therapy. On the second day of admission, repeat testing showed cyanide ion concentrations in the blood and gastric fluid were 0.1 μg/mL and 0.2 μg/mL, respectively. By the fourth day of treatment, the patient’s consciousness gradually cleared. After 1 week of treatment, cyanide ion tests in both blood and gastric fluid turned negative, and the patient was discharged after recovery. Follow-up examinations conducted 1 week and 2 months after discharge indicated that all functions had essentially returned to normal, with no residual sequelae. The flowchart of the patient’s treatment course is presented below (Figure 1).

Upon presentation with cardiac arrest, deep coma, severe lactic acidosis, and elevated central venous oxygenation, the differential diagnosis included conditions that impair cellular oxygen utilization or cause profound tissue hypoxia. These included cyanide poisoning, hydrogen sulfide poisoning, carbon monoxide poisoning, severe septic shock with microcirculatory dysfunction, and mitochondrial toxins such as metformin or sodium azide. Carbon monoxide poisoning was ruled out by normal carboxyhemoglobin levels. Septic shock was considered less likely due to the absence of fever or identifiable infection source, and the rapid progression to cardiac arrest without prior hypotension. The patient’s history of access to potassium ferrocyanide and its potential thermal decomposition to cyanide strongly supported cyanide poisoning as the leading diagnosis, subsequently confirmed by GC-MS.

### 2.1 Patient perspective

After regaining consciousness on day 4 of hospitalization, the patient provided a detailed account of the poisoning event. He reported that approximately 6 months prior to admission, he had placed 30 g of purchased potassium ferrocyanide in a beaker and heated it with a blowtorch. After observing the pale yellow powder turn black, he stored it at home. On the day of admission, he dissolved the black powder in 50 mL of water and ingested it orally, losing consciousness approximately 15 s later. He had no memory of the subsequent convulsions, cardiopulmonary resuscitation, or endotracheal intubation. Upon learning of the severity of his condition and the intensive treatments he had received, he expressed surprise at his survival. He had no prior psychiatric diagnosis or history of self-harm before this event. The patient was discharged after 1 week of treatment and was instructed to attend regular follow-up visits. Follow-up examinations at 1 week and 2 months after discharge showed that all organ functions had essentially returned to normal, with no residual sequelae.

## 3 Discussion

### 3.1 Presumed cause of poisoning

Potassium ferrocyanide, also known as yellow prussiate of potash, has the chemical formula K_4_[Fe(CN)_6_]. It appears as a yellow crystalline powder that forms pale yellow aqueous solutions. This compound is highly stable and exhibits very low toxicity. It is commonly used as an anti-caking agent in table salt, a pigment, and a food additive, and is considered safe and non-toxic within established food safety limits. However, potassium ferrocyanide is not safe. While it remains stable and resistant to decomposition at temperatures below 400 °C—a key characteristic enabling its use as a salt additive—it can decompose under high-temperature conditions to form potassium cyanide. This decomposition may have been the cause of potassium cyanide poisoning in this patient.

$$3K_{4}\left[\text{Fe}{\left(\text{CN}\right)}_{6}\right]==\Delta ==12\text{ KCN}+{\text{Fe}}_{3}C+5\;C+3\;N_{2}↑$$

### 3.2 Challenges in early diagnosis and alternative indicators

The “gold standard” diagnosis for cyanide poisoning relies on GC-MS, but this method is time-consuming and not conducive to early intervention. An arterial-venous oxygen concentration difference (P(a-v)O_2_) <4% and/or a significantly reduced arterial-venous oxygen partial pressure difference can aid in the early diagnosis of cyanide poisoning (Johnson and Mellors, 1988). However, its diagnostic efficacy decreases during cardiopulmonary resuscitation and with high-concentration oxygen inhalation. In this case, after initial resuscitation (ICU admission), the arterial PaO_2_ was 279.4 mmHg while PvO_2_ was abnormally elevated to 116.3 mmHg under FiO_2_ 100%, rendering P(a-v)O_2_ less reliable.

These observations, while derived from a single case, suggest that the combination of markedly elevated lactate and elevated PvO_2_ may serve as a potential bedside clue to raise suspicion of disorders of cellular oxygen utilization, including cyanide poisoning. However, this hypothesis requires validation in larger series. We emphasize that these are not validated diagnostic markers but rather hypothesis-generating observations to be considered in clinical context.

### 3.3 Dynamic indicators for assessing treatment response

The core pathogenic mechanism of cyanide lies in its rapid inhibition of cellular aerobic respiration. After entering the human body, cyanide ions (CN^−^) diffuse via the bloodstream to tissues throughout the body and enter the mitochondria of cells. There, they bind irreversibly to cytochrome c oxidase (Complex IV) at the terminal end of the respiratory chain, completely blocking electron transfer and ATP synthesis. This renders cells incapable of utilizing oxygen even in its presence, leading to a state of “intracellular asphyxiation.” Consequently, severe lactic acidosis is triggered, while the generation of carbon dioxide is reduced (Hall and Rumack, 1986; Lachowicz et al., 2024). When treatment is effective and mitochondrial function recovers, aerobic metabolism restarts, and CO_2_ production subsequently increases (Baud et al., 2018). In this case, 1 hour after treatment initiation, the patient’s PaCO_2_ showed no significant change (decreasing minimally from 45.4 mmHg to 44.5 mmHg), the patient’s PvCO_2_ increased from 44.5 mmHg to 72.7 mmHg, while the lactate level began to decrease. This observation raises the hypothesis that a rise in PvCO_2_ accompanied by a decrease in lactate may reflect the restoration of mitochondrial function and aerobic metabolism during effective treatment. However, we acknowledge that changes in PvCO_2_ in this case could have been influenced by multiple simultaneous interventions, including adjustments in mechanical ventilation (which directly affects PaCO_2_ and consequently PvCO_2_), administration of sodium bicarbonate (which generates CO_2_), improvement in systemic perfusion with vasoactive agents, and the effects of CRRT on acid-base balance and CO_2_ removal via the circuit. Therefore, the observed PvCO_2_ rise should be interpreted as a hypothesis-generating observation rather than a validated therapeutic marker. Future studies with frequent, paired arterial and venous blood gas sampling and controlled ventilation settings are needed to evaluate its utility.

### 3.4 The supportive role of blood purification within multimodal management

The mainstay of treatment for confirmed or strongly suspected cyanide poisoning is the timely administration of specific antidotes, including hydroxocobalamin and sodium thiosulfate. Current toxicology guidelines recommend empiric antidote administration when cyanide poisoning is suspected based on clinical presentation (e.g., coma, lactic acidosis, cardiovascular collapse), without waiting for confirmatory laboratory results (Lavonas et al., 2023; Leybell et al., 2025). Hydroxocobalamin is generally preferred due to its favorable safety profile, while sodium thiosulfate alone is slower-acting and often used as an adjunct.

In the present case, however, standard antidote therapy was not immediately available. Hydroxocobalamin was not stocked in our hospital, and the typical 12.5 g dose of sodium thiosulfate was also out of stock at the time of admission. A supply had to be transferred from another hospital. During the waiting period for the antidote to arrive, the patient received aggressive multimodal supportive care, including mechanical ventilation, vasoactive support, sodium bicarbonate, and early continuous venovenous hemofiltration. By the time a limited quantity of sodium thiosulfate (0.64 g, approximately 5% of the standard dose) became available, the patient’s hemodynamic status and metabolic acidosis had already substantially improved. Therefore, only this low dose was administered as a cautious adjunct rather than as definitive therapy.

This experience highlights that in settings where specific antidotes are unavailable or delayed, early and aggressive supportive care—particularly the rapid correction of life-threatening metabolic acidosis with blood purification—may play a critical role in bridging the patient until spontaneous elimination or until antidotes can be obtained. Blood purification technologies offer advantages in poisoned patients, including rapid removal of toxins (or their metabolites), improvement of organ function, maintenance of internal environment stability, and high safety (Patel and Bayliss, 2015; Yaxley and Scott, 2022). In the case reported by Liu et al., a patient who ingested sodium ferrocyanide and methanol began receiving plasma exchange and CRRT 2 h after ingestion; blood concentrations of the toxins decreased significantly, and the patient eventually regained consciousness and recovered (Liu et al., 2015). In our patient, blood purification likely contributed by rapidly correcting acidosis and removing potential cyanide or its metabolites, but it was one component of a successful multimodal strategy rather than the sole decisive intervention.

We acknowledge that if full-dose hydroxocobalamin or sodium thiosulfate had been available and administered earlier, the clinical course might have been different. Nonetheless, the favorable outcome in this case—achieved without standard antidote dosing—underscores the potential value of early blood purification as a supportive measure when antidotes are not readily accessible.

### 3.5 Limitations

This study is based on a single case report, and our inferences lack sufficient data validation; part of the retrospective data was incompletely recorded; moreover, there was a lack of more frequent monitoring of toxicant concentrations. The observed changes in PvCO_2_ may be confounded by multiple factors as discussed, and causality with treatment effect cannot be established. No comparison was made with standard full-dose antidote therapy (e.g., hydroxocobalamin), which is considered first-line in many settings; therefore, the relative contribution of blood purification versus other interventions remains uncertain.

## 4 Conclusion

The success rate of resuscitation for acute lethal cyanide poisoning is low. This case demonstrates that a multimodal approach—comprising early mechanical ventilation, hemodynamic support, bicarbonate therapy, gastric lavage, and continuous blood purification—can lead to favorable outcome even in the absence of full-dose antidote. Our observations regarding elevated PvO_2_ as a diagnostic clue and the dynamic change of PvCO_2_ with lactate as a potential treatment-response indicator are hypothesis-generating and require further validation. This report provides useful clinical insights for managing severe cyanide poisoning and may encourage future research into the role of early blood purification and bedside monitoring parameters.

## Data Availability

The original contributions presented in the study are included in the article/supplementary material, further inquiries can be directed to the corresponding author.
